# Selenium-enriched *Cardamine violifolia* improves growth performance with potential regulation of intestinal health and antioxidant function in weaned pigs

**DOI:** 10.3389/fvets.2022.964766

**Published:** 2022-08-11

**Authors:** Dan Wang, Yue Zhang, Qinliang Chen, Yanling Kuang, Jiajun Fan, Xiao Xu, Huiling Zhu, Qingyu Gao, Shuiyuan Cheng, Xin Cong, Yulan Liu

**Affiliations:** ^1^Hubei Key Laboratory of Animal Nutrition and Feed Science, Wuhan Polytechnic University, Wuhan, China; ^2^Enshi Se-Run Material Engineering Technology Co., Ltd., Enshi, China; ^3^National R&D Center for Se-rich Agricultural Products Processing, School of Modern Industry for Selenium Science and Engineering, Wuhan Polytechnic University, Wuhan, China

**Keywords:** *Cardamine violifolia*, piglets, Selenium, growth performance, antioxidant capacity, intestinal morphology, selenoprotein

## Abstract

This study was conducted to evaluate the effects of different Selenium (Se) sources on growth performance, intestinal function and antioxidant status of weaned piglets. A total of 300 weaned pigs were randomly allocated to 5 treatment groups with 5 replicates of 12 pigs/pen. The control group was corn-soybean basal diet without any additional Se supplement. The experimental diets were supplemented with 0.3 mg/kg of Se from sodium selenite (SS), Se-enriched yeast (SEY), Se-enriched *Cardamine violifolia* (SEC) and 0.3+0.3 mg/kg of Se from SEY and SEC, respectively. The trial lasted for 4 weeks. The results showed that diets supplementation with SEY, SEC or SEY+SEC could improve average daily gain and reduce feed/gain ratio during the entire study. Compared with the control group, SEC or SEY+SEC improved intestinal morphology, indicated by greater villus height and villus height/ crypt depth ratio. In addition, SEC or SEY+SEC also increased maltase and lactase activities as well as tight junction protein expression. Different Se sources decreased malondialdehyde (MDA) concentration and improved superoxide dismutase (SOD) activity in serum. In the jejunum, SEY or SEC reduced MDA concentration and increased total antioxidant capacity (T-AOC) compared with the control group. Moreover, SEY+SEC increased the antioxidant parameters including SOD and T-AOC in the jejunum. Dietary SEY or SEC supplementation significantly increased the mRNA expression of selenoproteins including thioredoxin reductase 1 (TXNRD1), selenoprotein I (SELENOI), selenoprotein S (SELENOS), and selenoprotein P (SELENOP) in the jejunum. In conclusion, organic Se sources, especially *Cardamine violifolia*, improve growth performance, potentially by regulating intestinal function, antioxidant capacity and selenoprotein expression in piglets.

## Introduction

Weaning is the most significant event in the life of pigs as they are subject to constant nutritional, psychological and environmental stresses during weaning, which can result in enormous economic losses to the swine industry. During the weanling period, stresses usually result in the perturbations in host physiology, and mucosal immune function with subsequent reduction in feed intake, occurrence of post-weaning diarrhea, and growth reduction in piglets (1). These metabolic disorders, induced by weanling stress, are associated with inflammatory response, and impairment of redox balance and intestinal health (2, 3). This is why there is a wide range of interests in the field of swine science and production in developing both management and feeding strategies to enhance intestinal health in weanling piglet.

Selenium (Se), an essential trace element, acts as a feedstuff additive that is widely used in animal production. Se exerts various functions such as anti-oxidation, immunity, growth, and thyroid hormone metabolism (4). The function of Se is mainly derived from its presence in selenocysteine, which constitute the active site of various selenoproteins (5). Growing evidence suggest that Se deficiency has been associated with poor immunity, various tissue damage as well as an increased risk of mortality in piglets (6, 7). Recently, many studies have shown that dietary Se supplementation alleviates immune stress, oxidative stress and heat stress in pigs (8–10). These clues suggest that Se may play an important role in attenuating weaning stress of piglets. The current recommendation of Se in swine diets is 0.30 mg/kg (NRC, 2012). Traditionally, Se is generally supplemented to pig diets as sodium selenite, an inorganic form. However, there has been increasing interest in organic Se such as Se-enriched yeast and Se-enriched plant in recent years because of its higher absorption and biological effectiveness and lower environmental pollution in animal production (11–13). Organic Se has also been reported to have higher antioxidant activity, whereas the inorganic form may act as a prooxidant and have toxic effects particularly at high levels (14).

*Cardamine violifolia* is a Se-tolerant species native to the highly seleniferous region of Enshi, China. It can accumulate Se content exceeding 700 mg/kg dry weight in the leaves and about 85% in the form of Se-enriched proteins. *Cardamine violifolia* has higher organic Se content than Se-enriched yeast, Se-enriched peanuts, or other Se-enriched products (15). Moreover, it has an edible history of several 100 years and is enriched in various nutrients, especially abundant proteins, vitamin C, and minerals. Emerging studies have shown that *Cardamine violifolia* exerts various beneficial effects, such as anti-oxidation, anti-fatigue, weight loss and anti-aging in brain (16–19). For example, *Cardamine violifolia* prevented high fat diet-induced obesity and metabolic disorders by ameliorating oxidative stress and inflammation (17). The edibility and Se-enriching ability of *Cardamine violifolia* display it as a potential source of Se supplementation for animal production. Currently, few studies had been conducted to explore the effect of *Cardamine violifolia* on growth performance and health status of weaned piglets.

In this study, we evaluated the effects of different Se sources on growth performance, intestinal function, and antioxidant capacity in weaned piglets. We showed that organic Se sources, especially *Cardamine violifolia* improved growth performance and health status in piglets after 4 weeks feeding. The mechanism of organic Se-promoting growth was to improve digestive and barrier function of small intestine, as well as antioxidant capacity in the body. This study could provide novel insights into the application of *Cardamine violifolia* for livestock industry.

## Materials and methods

### Experimental design and sample collection

All animal experiments and procedures were approved by Animal Care and Use Committee of Wuhan Polytechnic University. A total of 300 weaned pigs (Duroc × Large White × Landrace; barrows; 9.70 ± 1.72 kg initial body weight; 35 ± 1 d of age), were randomly allocated to 5 treatment groups with 5 replicates of 12 pigs/pen (6 barrows and 6 gilts). Experimental diets were formulated to provide nutrient composition above NRC (2012) recommendation diets (Table 1). The control group was fed a Se-deficient basal diet. The experimental treatments were supplemented with 0.3mg Se/kg feed from sodium selenite (SS), Se-enriched yeast (SEY), Se-enriched *Cardamine violifolia* (SEC), and SEY+SEC (0.3+0.3mg Se/kg) in the basal diet, respectively. The actual level of Se in the diet of each group is shown in Supplementary Table 1. Feed and water were provided *ad libitum* during the study. The weight and feed intake were measured on day 1, 14, and 28 for calculation of average daily gain, average daily feed intake, and the feed/gain ratio. Dried *Cardamine violifolia* powder (1,430 mg/kg total Se content) was obtained from Enshi Se-Run Material Engineering Technology Co. (Enshi, China).

**Table 1:** Ingredient composition of diets (as-fed basis)^1^.

**Items**	**Content (g/kg)**
Ingredients	
Maize	584.0
Soybean meal (44%CP)	222.7
Fermented soybean	115.8
Fish meal	19.5
Dicalcium phosphate	17.3
Fat powder	9.6
Sugar	6.6
Limestone	6.2
Whey power	6.0
Salt	4.8
L-Lysine, HCL (78.8% Lysine)	3.8
Acidifier^2^	2.0
DL-Methionine (99 % Methionine)	0.7
Vitamin and mineral premix^3^	1.0
Nutrient composition	
Digestible energy^4^ (MJ kg^−1^)	13.5
Crude protein^5^	202
Calcium^5^	9.0
Total phosphorus^5^	7.0
Total lysine^4^	13.0
Total methionine + cysteine^4^	7.0

^1^The basal diet was formulated in accordance with NRC (2012) requirements for all nutrients.

^2^A compound acidifier including lactic acid and phosphoric acid, provided by Shanghai Aodeng Biotechnology Company, Shanghai, China.

^3^Vitamin and mineral premix provided the following amounts per kg of complete diet: retinol acetate, 2,700 μg; cholecalciferol, 62.5 μg; DL-α-tocopheryl acetate, 20 mg; menadione, 3 mg; vitamin B_12_, 18 mg; riboflavin, 4 mg; niacin, 40 mg; pantothenic acid, 15 mg; choline chloride, 400 mg; folic acid, 700 mg; thiamin, 1.5 mg; pyridoxine, 3 mg; biotin, 100 mg; Zn, 80 mg (ZnSO_4_·7H_2_O); Mn, 20 mg (MnSO_4_·5H_2_O); Fe, 83 mg (FeSO_4_·H_2_O); Cu, 25 mg (CuSO_4_·5H_2_O); I, 0.48 mg (KI); Se, 0.30 mg (sodium selenite, Se-enriched yeast or Se-enriched Cardamine violifolia).

^4^Calculated.

^5^Analyzed.

On day 14 and 28, blood samples were collected into heparinized vacuum tubes. The samples were then centrifuged to separate plasma and stored at −80 °C for analysis. After 28-day feeding trial, 6 pigs were selected from each group and were slaughtered with sodium pentobarbital (80 mg kg/ BW). The abdomen was immediately opened. The jejunum was removed, and flushed with ice-cold phosphate buffered saline (PBS). The 3-cm long segment of intestine was harvested from the mid-jejunum. About 10-cm middle portions of jejunum were opened longitudinally and cleaned with PBS. Mucosal samples were collected by scratching with a glass slide from the connective tissue, and snap-frozen in liquid nitrogen and then stored at −80 °C until further analysis.

### Intestinal histology

Jejunal samples were fixed with 4% paraformaldehyde solution and then embedded in paraffin. Three cross sections (5 μm) of each intestinal segment were stained with hematoxylin and eosin (H&E). Ten well-oriented, intact villi and their associated crypts from each segment were used to measure villus height and crypt depth using a light microscope equipped with a calibrated eyepiece graticule (BioScan Optimetric, BioScan Inc., Edmonds, WA, USA). The ratio of villus height and crypt depth was calculated.

### Serum and intestinal antioxidant enzymes

Total antioxidant capacity (T-AOC), superoxide dismutase (SOD) activity, and malondialdehyde (MDA) concentration in serum and intestinal mucosa were determined using the commercial kits (T-AOC, A015-1; SOD, A001-1; MDA, A003-1) obtained from Nanjing Jiancheng Bioengineering Institute (Nanjing, China) according to the manufacturer's procedures. Total protein concentration in intestinal mucosa was measured according to the instructions of the bicinchoninic acid protein assay kit (Beyotime, Beijing, China). T-AOC and SOD activity were expressed as units per milliliter (U/mL) in serum and units per milligram (U/mg) of total protein in the jejunum, respectively. MDA concentration was expressed as nanomole per milliliter (nmol/mL) in serum and nanomole per milligram (nmol/mg) of total protein in the jejunum, respectively.

### Intestinal mucosal disaccharidase activities

The activities of lactase, sucrose, and maltase in intestinal mucosa were measured according to the method of Liu et al. (20) using a glucose kit (lactase, #082-1; sucrose, #082-2; maltase, #082-3) purchased form Nanjing Jiancheng Bioengineering Institute (Nanjing, China). Enzyme activity was expressed as U/mg protein in the jejunum.

### RNA extraction and quantitative RT-PCR

Total RNA was extracted from jejunal mucosa using TRIzol reagent (Invitrogen), according to the manufacturer's instructions. Total RNA (1 μg) was reverse transcribed with PrimeScript RT reagent kit with gDNA eraser (TaKaRa, #RR047A) for quantitative RT-PCR with SYBR Premix Ex Taq (Tli RNase H Plus) qPCR kit (TaKaRa, #RR420A) and 7500 Real-Time PCR system (Applied Biosystems). The relative mRNA abundance of the target genes was standardized with β-actin as the invariant control and was analyzed by the 2^−Δ*ΔCT*^ method of Livak and Schmittgen. Primer sequences are given in Supplementary Table 2.

### Statistical analysis

All data were subjected to one-way ANOVA analysis using SPSS version 22.0 (SPSS Inc., Chicago, IL, USA) appropriate for a factorial arrangement of treatments in a randomized complete block design. The differences among group means were compared using least significant difference (LSD) multiple comparison based on the variance derived from ANOVA. Pen was used as the experimental unit for the performance data, whereas individual pig data were used as the experimental unit for intestinal morphology and enzyme-specific activity. *P* < 0.05 was considered to be statistically significant. All data are presented as mean ± SEM.

## Results

### Growth performance

The data of growth performance are presented in Table 2. After 4 weeks feeding trial, final body weight of all Se supplemented groups was significantly higher compared with the control group (*P* < 0.05). Compared with the control group, SS group displayed higher average daily gain and lower feed/gain ratio during weeks 1–2 (*P* < 0.05); SEY supplementation significantly decreased feed/gain ratio during weeks 1–2 (*P* < 0.05) and increased average daily gain over the entire study (*P* < 0.05); Diets supplemented with SEC or SEY+SEC significantly increased average daily gain and decreased feed/gain ratio during weeks 3–4 and over the entire study (*P* < 0.05). Moreover, the feed/gain ratio of SEC group was significantly lower compared with other four groups (*P* < 0.05). No difference was observed in average daily feed intake and diarrhea rate among the treatments.

**Table 2:** Effects of different Se sources on growth performance of weaned piglets^1^.

**Item**	**Diets** ^**2**^					**SEM^3^**	***P* values^4^**
	**Ctrl**	**SS**	**SEY**	**SEC**	**SEY+SEC**		
1 day BW^5^(kg)	9.71	9.71	9.69	9.67	9.71	0.02	0.367
14 day BW(kg)	13.80^b^	14.31^a^	14.06^ab^	14.44^a^	14.32^a^	0.16	0.066
28 day BW(kg)	20.11^b^	20.81^a^	20.91^a^	21.15^a^	21.21^a^	0.18	0.005
Weeks 1–2							
ADG^5^(g/d)	302^b^	334^a^	317^ab^	336^a^	321^ab^	9.20	0.101
ADFI^5^(g/d)	595	614	589	577	603	0.02	0.623
Feed/gain ratio(g/g)	1.99^a^	1.84^bc^	1.87^b^	1.72^c^	1.88^ab^	0.04	0.004
Weeks 3–4							
ADG(g/d)	459^b^	458^b^	483^ab^	492^a^	496^a^	10.64	0.054
ADFI(g/d)	793	793	810	776	804	0.02	0.732
Feed/gain ratio(g/g)	1.72^a^	1.74^a^	1.68^ab^	1.57^c^	1.61^bc^	0.03	0.005
Weeks 1–4							
ADG(g/d)	381^b^	395^ab^	399^a^	410^a^	409^a^	6.75	0.049
ADFI(g/d)	690	699	694	671	699	0.01	0.656
Feed/gain ratio(g/g)	1.82^a^	1.77^ab^	1.74^ab^	1.63^c^	1.70^bc^	0.02	0.001
Diarrhea rate(%)	3.72	4.24	4.03	4.46	2.95	0.49	0.28

^1^Data are means of 5 replicate pens of 12 pigs each replicate.

^2^Ctrl, control group; SS, sodium selenite; SEY, Se-enriched yeast, SEC, Se-enriched Cardamine violifolia; SEY+SEC, Se-enriched yeast + Se-enriched Cardamine violifolia.

^3^Standard error of the mean.

^4^Probability for contrast of 5 treatments by LSD's Multiple Comparison. ^a, b, c^Means within a row with different letters were significantly different compared to the other groups (P < 0.05).

^5^BW, body weight; ADG, average daily gain; ADFI, average daily feed intake.

### Intestinal morphology

**Figure 1:**
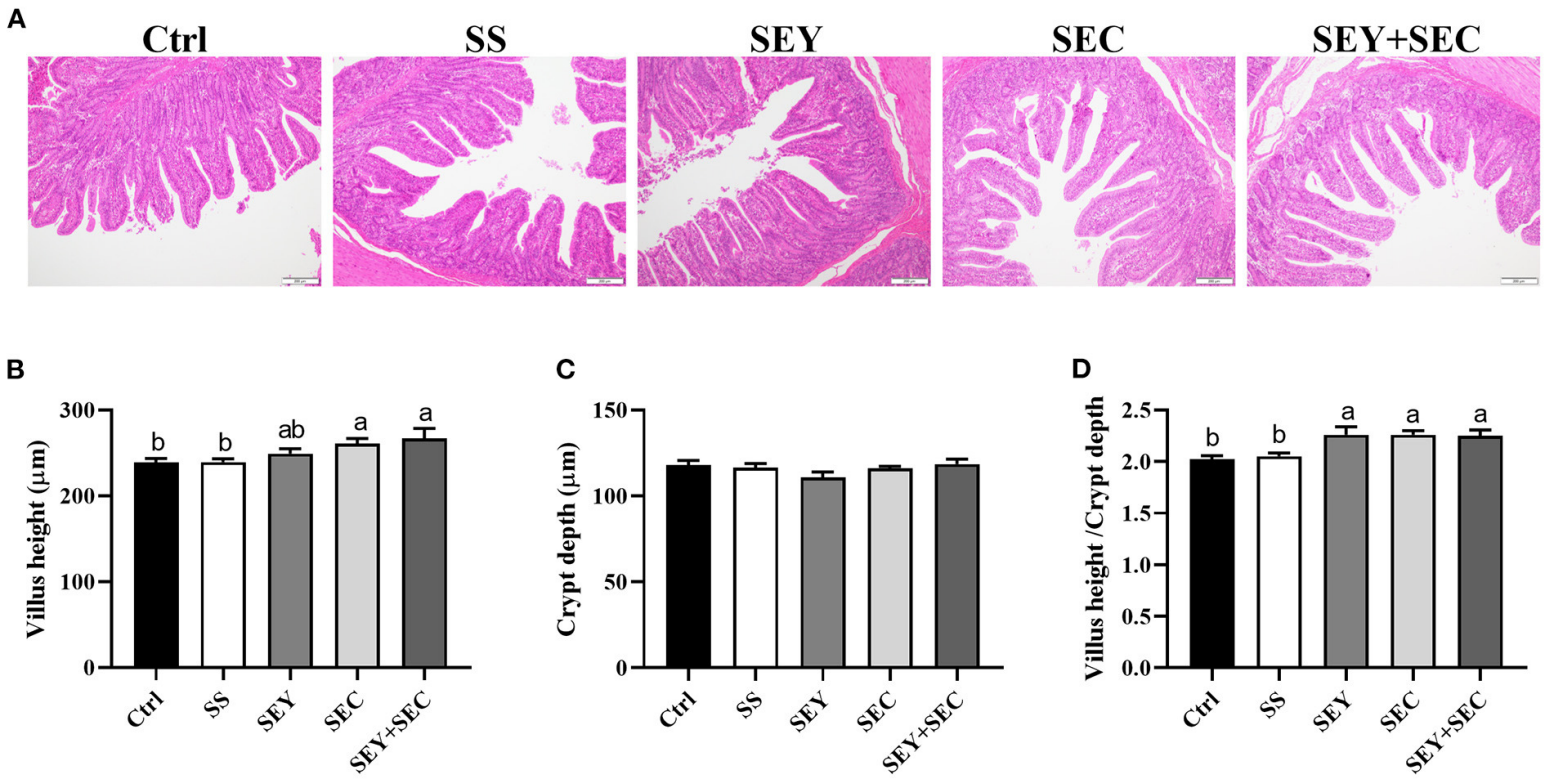
Effects of different Se sources on jejunal morphology in piglets. **(A)** The histopathology of jejunum by H&E staining, Scale bar, 100 μm; **(B–D)** The villus height **(B)**, crypt depth **(C)**, and villus height/ crypt depth ratio **(D)** in the jejunum. Values are means ± SEM, *n* = 4–6. ^a, b^Means without a common letter differ, *P* < 0.05. Ctrl, control group; SS, sodium selenite; SEY, Se-enriched yeast, SEC, Se-enriched *Cardamine violifolia*; SEY+SEC, Se-enriched yeast + Se-enriched *Cardamine violifolia*.

As shown in Figure 1, dietary SEC or SEY+SEC supplementation significantly increased villus height and the ratio of villus height: crypt depth (*P* < 0.05) compared with the control group in the jejunum (Figures 1A,B,D). Compared with the control group, SEY group also displayed higher the ratio of villus height: crypt depth (*P* < 0.05) in the jejunum (Figures 1A,D), whereas there was no difference between SS group and control group (Figures 1A,D). Supplementation with different Se sources exerted no effect on crypt depth in the jejunum (Figures 1A,C).

### Intestinal digestive enzyme activity

**Figure 2:**
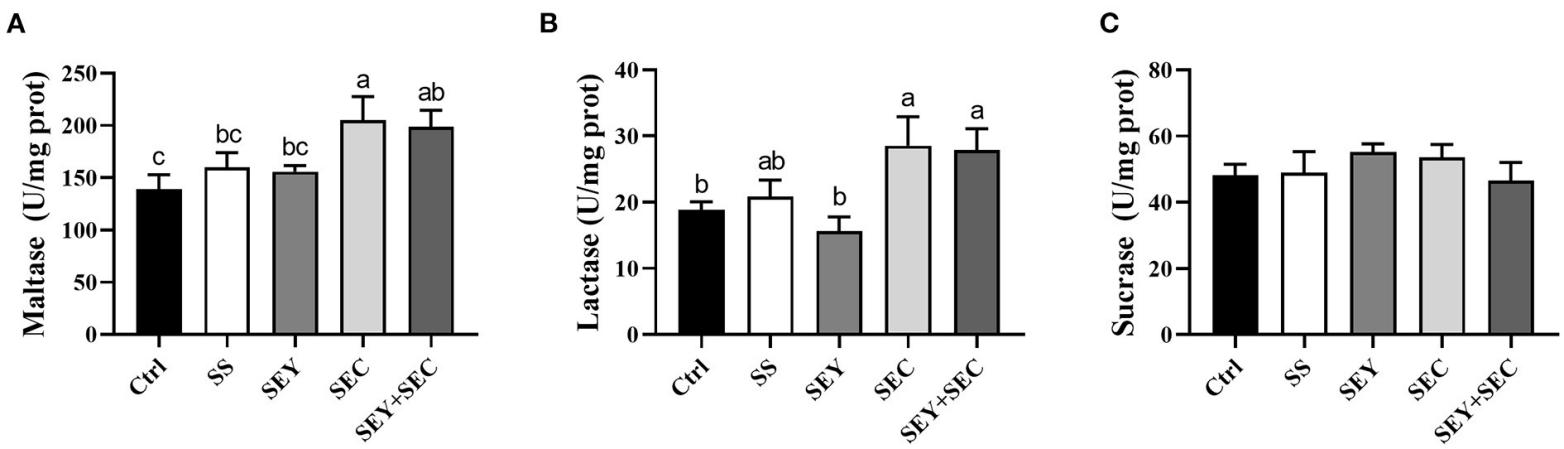
Effects of different Se sources on the disaccharidase activities of jejunum in piglets. **(A–C)** The activity of maltase **(A)**, lactase **(B)**, and sucrase **(C)** in the jejunum. Values are means ± SEM, *n* = 6. ^a, b, c^Means without a common letter differ, *P* < 0.05. Ctrl, control group; SS, sodium selenite; SEY, Se-enriched yeast, SEC, Se-enriched *Cardamine violifolia*; SEY+SEC, Se-enriched yeast + Se-enriched *Cardamine violifolia*.

As shown in Figure 2, dietary SEC or SEY+SEC supplementation significantly increased maltase and lactase activities (*P* < 0.05) compared with the control group (Figures 2A,B), but did not affect sucrase activity in the jejunum (Figure 2C). SS or SEY has no effect on maltase, lactase, and sucrase activities in the jejunum (Figures 2A–C).

### Intestinal tight junction protein expression

**Figure 3:**
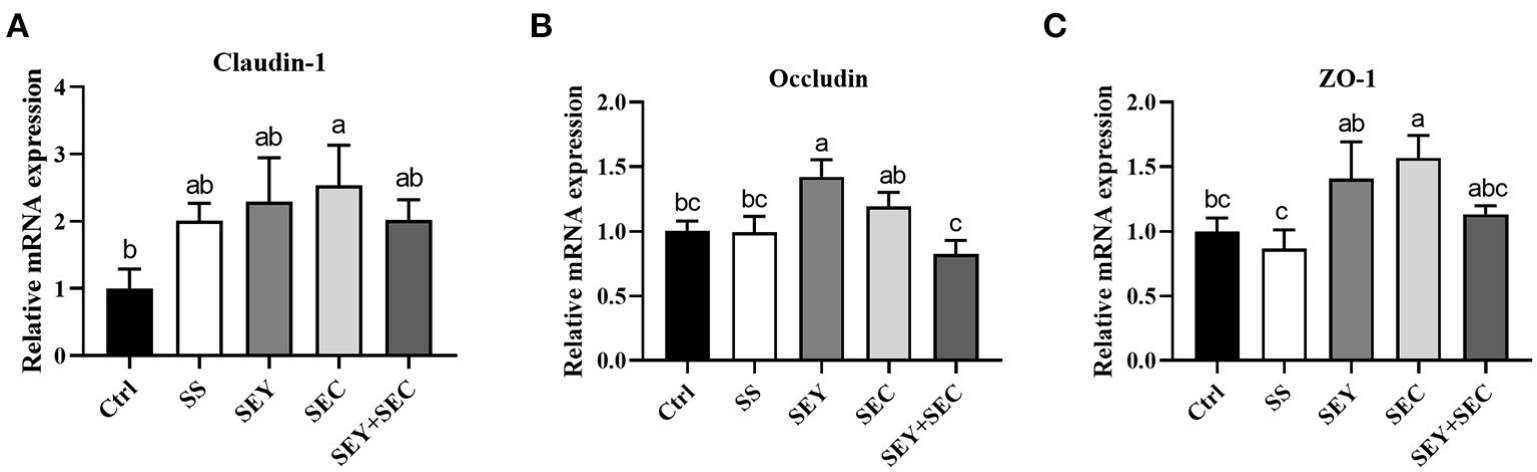
Effects of different Se sources on tight junction protein mRNA expression in the jejunum. **(A–C)** The mRNA expression of claudin-1 **(A)**, occludin **(B)**, and ZO-1 **(C)** in the jejunum. Values are means ± SEM, *n* = 6. ^a, b, c^Means without a common letter differ, *P* < 0.05. Ctrl, control group; SS, sodium selenite; SEY, Se-enriched yeast, SEC, Se-enriched *Cardamine violifolia*; SEY+SEC, Se-enriched yeast + Se-enriched *Cardamine violifolia*.

As shown in Figure 3, dietary SEC supplementation significantly increased the mRNA expression of claudin-1 and ZO-1 (*P* < 0.05) compared with the control group in the jejunum (Figures 3A,C); SEY enhanced the mRNA expression of occludin (*P* < 0.05) (Figure 3B); SS or SEY+SEC has no effect on claudin-1, occludin and ZO-1 mRNA expression in the jejunum (Figures 3A–C).

### Serum and jejunal antioxidant parameters

**Figure 4:**
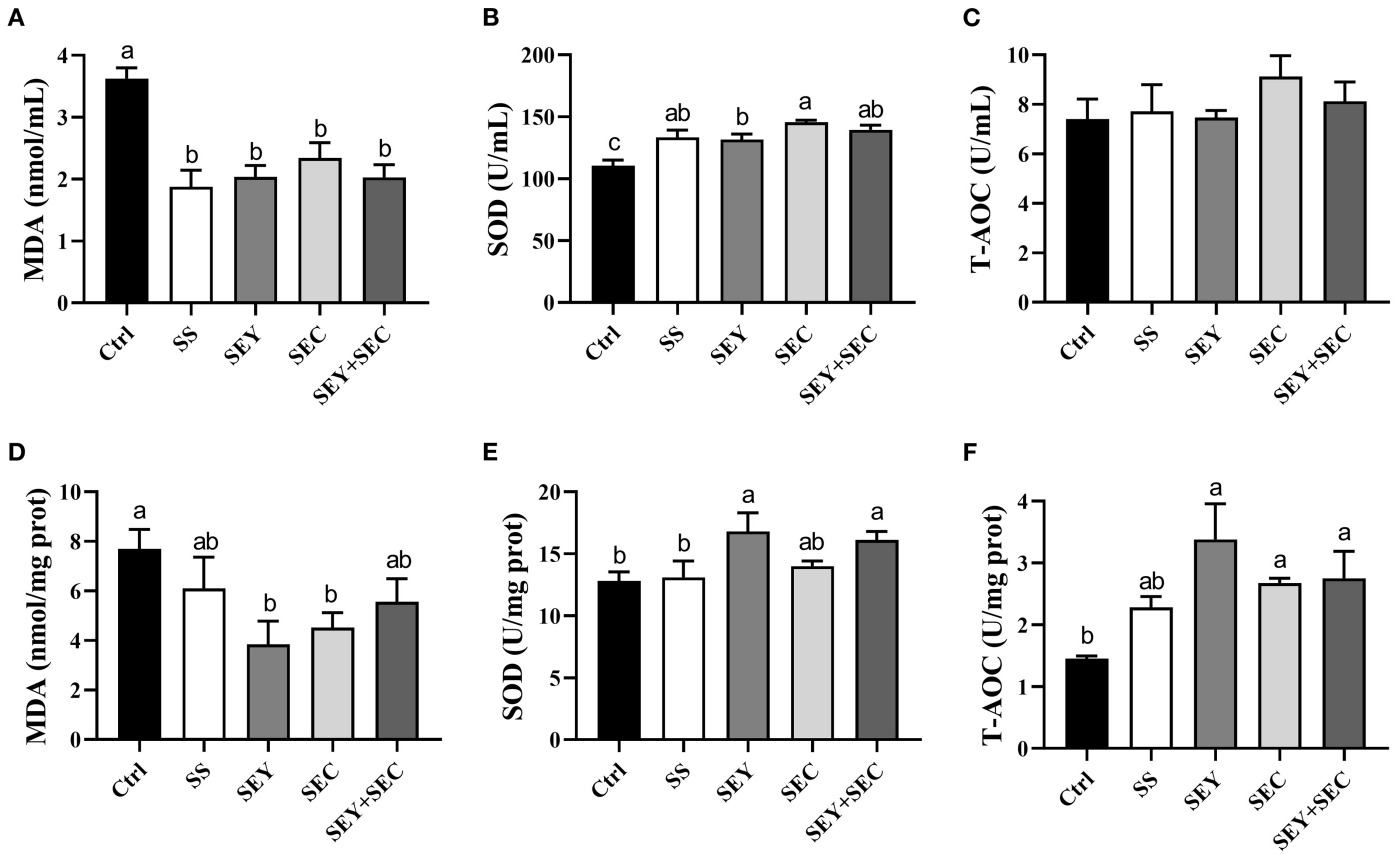
Effects of different Se sources on the antioxidant parameters in serum and jejunum. **(A–C)** MDA concentration **(A)**, SOD activity **(B)** and T-AOC **(C)** in serum; **(D–F)** MDA concentration **(D)**, SOD activity **(E)**, and T-AOC **(F)** in the jejunum. Values are means ± SEM, *n* = 6. ^a, b, c^Means without a common letter differ, *P* < 0.05. MDA, malondialdehyde; SOD, superoxide dismutase; T-AOC, total antioxidant capacity. Ctrl, control group; SS, sodium selenite; SEY, Se-enriched yeast, SEC, Se-enriched *Cardamine violifolia*; SEY+SEC, Se-enriched yeast + Se-enriched *Cardamine violifolia*.

As shown in Figure 4, supplementation with different Se sources significantly reduced MDA concentration (*P* < 0.05) and increased SOD activity (*P* < 0.05) in serum of piglets compared with the control group (Figures 4A,B), while did not affect T-AOC (Figure 4C). Interestingly, SOD activity of SEC group in serum was also higher than that of SEY group (Figure 4B). In jejunal mucosa, dietary SEY supplementation significantly reduced MDA concentration (*P* < 0.05) and increased SOD activity and T-AOC (*P* < 0.05) compared with the control group (Figures 4D–F); SEC group also had lower MDA concentration (*P* < 0.05) and higher T-AOC (*P* < 0.05) in the jejunum (Figures 4D,F). SEY+SEC supplementation significantly increased SOD activity and T-AOC (*P* < 0.05) in the jejunum (Figures 4E,F). However, no difference was observed in these antioxidant parameters between SS group and the control group in the jejunum (Figures 4D–F).

### Intestinal selenoprotein mRNA expression

**Figure 5:**
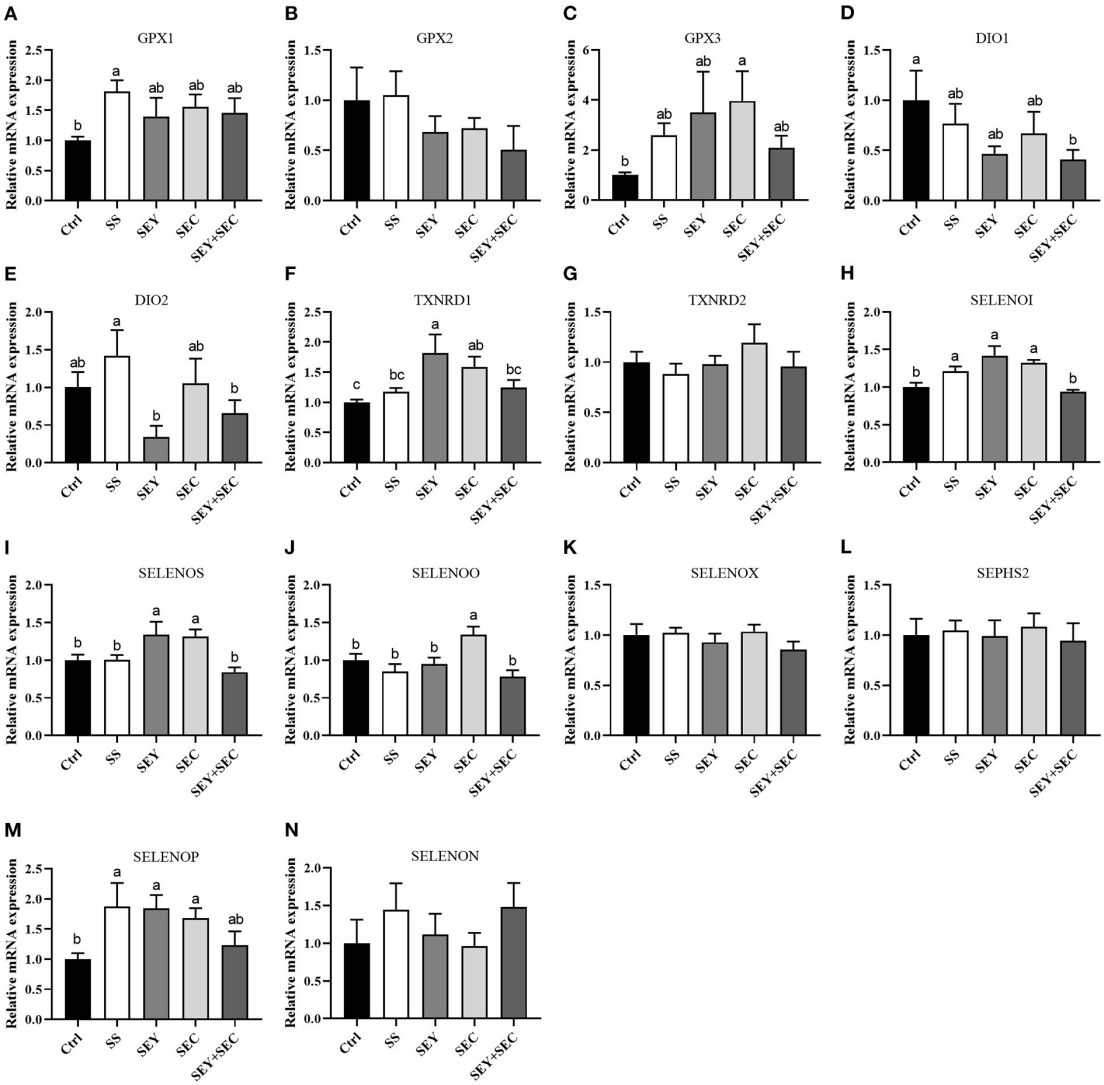
Effects of different Se sources on the mRNA expression of selenoproteinss in the jejunum. **(A–N)** The mRNA expression of selenoproteins, including glutathione peroxidase 1 (GPX1), GPX2, GPX3, iodothyronine deiodinase 1 (DIO1), DIO2, thioredoxin reductase 1 (TXNRD1), TXNRD2, selenoprotein I (SELENOI), selenoprotein S (SELENOS), selenoprotein O (SELENOO), selenoprotein X (SELENOX), selenophosphate synthetase 2 (SEPHS2), selenoprotein P (SELENOP) and selenoprotein N (SELENON) in the jejunum. Values are means ± SEM, *n* = 6. ^a, b, c^Means without a common letter differ, *P* < 0.05. Ctrl, control group; SS, sodium selenite; SEY, Se-enriched yeast, SEC, Se-enriched *Cardamine violifolia*; SEY+SEC, Se-enriched yeast + Se-enriched *Cardamine violifolia*.

As shown in Figure 5, SS group had higher mRNA expression of glutathione peroxidase 1(GPX1), selenoprotein I (SELENOI) and selenoprotein P (SELENOP) (*P* < 0.05) compared with the control group in the jejunum (Figures 5A,E,H,M). Dietary SEY supplementation significantly increased the mRNA expression of thioredoxin reductase 1 (TXNRD1), SELENOI, selenoprotein S (SELENOS) and SELENOP (*P* < 0.05) compared with the control group (Figures 5F,H,I). The mRNA expression of GPX3, TXNRD1, SELENOI, SELENOS, selenoprotein O (SELENOO) and SELENOP were higher (P < 0.05) in SEC group than that of control group in the jejunum (Figures 5C,F,H–J,M). However, dietary SEY+SEC supplementation significantly decreased the mRNA expression of iodothyronine deiodinase 1 (DIO1) (*P* < 0.05) in the jejunum (Figure 5D). Supplementation with different Se sources did not affect the mRNA expression of GPX2, TXNRD2, selenoprotein X (SELENOX), selenophosphate synthetase 2 (SEPHS2) and selenoprotein N (SELENON), compared with the control group (Figures 5B,G,K,L,N).

## Discussion

Se, as a component of some antioxidant enzymes and proteins involved in the subsequent scavenge progress, participates in antioxidant system regulation. Previous studies in pigs revealed that Se deficiency from marginal to moderate led to growth retardation, immunity disorder, gastrointestinal impairment, and oxidative stress (21–23). In our study, the effects of different Se sources on growth performance of weaned piglets were assessed. Supplementation with different Se sources has beneficial effects on growth performance, including increased body weight gain and decreased feed/gain ratio after 4 weeks feeding. These data confirm that Se is an essential trace element in pig nutrition. More importantly, the feed/gain ratio of SEC group was significantly lower than that of SS or SEY group, indicating that SEC had better growth promotion effects compared with other Se sources. The possible explanation is that *Cardamine violifolia* contains not only organic Se but also other nutrients, such as soluble sugars, amino acids and sulfo-compounds, which are generally beneficial for animal health. It was worth noting that no difference was observed in growth performance of weaned piglets between SEC and SEC+SEY group, suggesting no synergistic combination effect between *Cardamine violifolia* and Se-enriched yeast. The data suggest that the form of Se has a greater impact on growth performance of piglets than the level of Se once Se meets adequate recommendations.

Villus height and crypt depth are commonly used as key indicators for evaluating intestinal integrity (20). Small intestine has a typical villus-crypt architecture, which plays critical roles in nutrient digestion and absorption. Our findings showed that villus height and villi height/ crypt depth ratio were significantly increased in SEC or SEY+SEC groups compared with the control group and SS group. Consistent with our findings, Yu et al. (17) reported that Se-enriched peptides from *Cardamine violifolia* significantly improved intestinal morphology, indicated by greater villus height and villus height/ crypt depth ratio. Se-enriched yeast also increased the ratio of villus height/crypt depth in small intestine, suggesting that organic Se had better roles in promoting intestinal development than inorganic Se. In addition, the activities of jejunal digestive enzymes including maltase and lactase were increased in SEC or SEY+SEC group. These data suggest that SEC promotes intestinal mucosal maturation and digestive function. Intestinal tight junctions are the key barriers that prevent the penetration by luminal bacteria and dietary allergens into the mucosa. In our study, SEY or SEC supplementation enhanced protein expression of occludin and claudin-1 in the jejunum, whereas SS had no effect. Yang et al. (24) reported that Se-enriched yeast also increased the expression of these tight junction genes, and antagonized intestinal barrier injury caused by ochratoxin A in broilers. Similarly, Se-enriched peptides from *Cardamine violifolia* were reported to upregulate ZO-1 and occludin expression in the ileum, and restored high fat diet-induced barrier permeability in mice (17). These data demonstrate that organic Se sources, especially *Cardamine violifolia*, improve growth performance by maintaining intestinal health in weaned piglets.

Redox imbalance is strongly correlated with animal growth and health (23, 25–27). It is widely accepted that proper Se intake can enhance antioxidant capacity in pigs (22, 28, 29). Antioxidant enzymes, such as SOD, are the vital component of antioxidant defense systems involved in scavenging reactive oxygen species and maintaining the redox equilibrium. MDA is a metabolite produced by lipid peroxidation and widely used as an indicator of oxidative stress (30). In the present study, regardless of sources, Se supplementation significantly reduced MDA concentration and increased SOD activity in serum, declaring that Se supplementation suppressed lipid peroxidation and promoted the antioxidant capacity. Furthermore, SEY or SEC group showed lower MDA concentration and higher SOD activity compared with the control group in the jejunum. Our results are in line with the previous study that SEY supplementation has the potential effects on ameliorating the deleterious impacts of oxidative stress injury (8). Recently, Se-enriched peptides from *Cardamine violifolia* is reported to ameliorate oxidative stress in the hippocampus of D-galactose-induced aging rats (18). Notably, SOD activity of SEY or SEY+SEC group was higher than that of SS group in the jejunum. Similarly, Cao et al. (28) showed that diets supplemented with selenomethionine (an organic Se) was more effective than sodium selenite to decrease MDA concentration in serum and organs. Together, these data suggest that organic Se has better antioxidant properties than its inorganic forms (31).

Se exhibits its antioxidant functions through its incorporation into selenoproteins, of which 25 have been identified in pigs (32). In our study, mRNA expression of 14 major selenoproteins was determined in the jejunum of weaned piglets. GPX is the first identified selenoprotein, which is involved in the catabolism of peroxides (9). Our data showed that GPX1 expression in SS group and GPX3 expression in SEC group were upregulated compared with the control group in the jejunum. However, dietary Se sources did not affect GPX2 mRNA expression. In fact, GPX2, which is abundant in the intestine, has been shown to be protective against oxidative stress during inflammation (33). Liu et al. (34) found that when the dietary Se level increased the GPX2 mRNA and GPX activities increased linearly in the intestine of pigs. The possible explanation is that the differences of Se sources and the duration of treatment lead to differences in GPX2 expression. DIO1 is a plasma membrane protein, and plays an important role in inflammation and immunity (35). Our data showed that the gene expression of DIO1 was decreased in SEY+SEC group, suggested that high level of Se in diets may reduce immunity in the body. TXNRD catalyzes the NADPH dependent reduction of thioredoxin and therefore plays an important role in antioxidant defense and redox regulation (36). In this study, the mRNA expression of TXNRD1 was increased in the jejunum by SEY or SEC supplement, but the mRNA expression of TXNRD2 was unchanged. Previous study also showed that TXNRD1 reduced the oxidative stress during inflammatory bowel disease as well as inhibited intestinal inflammation (37). Other selenoprotein genes, including SELENOI, SELENOS, and SELENON, are endoplasmic reticulum proteins that seem to be involved in redox balance and the unfolded protein response (38). Our data showed that the gene expression of SELENOI and SELENOS were increased in SEY or SEC group. Ding et al. (39) showed that maternal 2-hydroxy-4-methylselenobutanoic acid supplementation significantly upregulated mRNA level of SELENOS in the jejunum of newborn and weaned piglets compared with the control group. Collectively, changes in selenoprotein genes induced by organic Se may enhance the antioxidant capacity in the intestine, and thus improve the growth performance of piglets.

## Conclusions

The present study demonstrates that Se-enriched *Cardamine violifolia* has better growth promotion effect than sodium selenite or Se-enriched yeast in the diet of weaned pigs. The supplementation of *Cardamine violifolia* in weaned pig diet can improve growth performance, intestinal function, antioxidant capacity, and selenoprotein expression at the level of 0.3 mg/kg. These results will also provide scientific evidences for the development of antioxidant drugs in the future as well as the application of *Cardamine violifolia* in piglets.

## Data availability statement

The original contributions presented in the study are included in the article/Supplementary material, further inquiries can be directed to the corresponding authors.

## Ethics statement

The animal study was reviewed and approved by Animal Care and Use Committee of Wuhan Polytechnic University.

## Author contributions

DW and YL designed the study, wrote the manuscript, and acquired funding. YZ, QC, YK, JF, QG, and XC conducted animal trial. XX, HZ, and SC analyzed the parameters and data. DW, XC, and YL read and approved the final version. All authors contributed to the article and approved the submitted version.

## Funding

This research was financially funded by the Project of Innovative Research Groups of the Natural Science Foundation of Hubei Province (No. 2019CFA015), National Natural Science Foundation of China (No. 32102566), and the Hubei Province's Key Project of Research and Development Plan (No. 2020BBA043).

## Conflict of interest

Authors YZ, QG, and XC were employed by Enshi Se-Run Material Engineering Technology Co., Ltd. The remaining authors declare that the research was conducted in the absence of any commercial or financial relationships that could be construed as a potential conflict of interest.

## Publisher's note

All claims expressed in this article are solely those of the authors and do not necessarily represent those of their affiliated organizations, or those of the publisher, the editors and the reviewers. Any product that may be evaluated in this article, or claim that may be made by its manufacturer, is not guaranteed or endorsed by the publisher.
