# Supplementary material for: Selenium-enriched Cardamine violifolia improves growth performance with potential regulation of intestinal health and antioxidant function in weaned pigs
Source: Front Vet Sci. 2022 Aug 11;9:964766. doi: 10.3389/fvets.2022.964766 (PMC9403540; doi:10.3389/fvets.2022.964766)
Supplement: Supplementary file 1 [file Table_1.docx]

Supplementary Material

# Supplementary Table 1. The level of Se in the diet of each group

| **Item** | **Diets** | | | | |
| --- | --- | --- | --- | --- | --- |
|  | **Ctrl** | **SS** | **SEY** | **SEC** | **SEY+SEC** |
| Se content (mg/kg) | 0.058 | 0.230 | 0.227 | 0.219 | 0.468 |

# Primer sequences used for real-time PCR

| **Gene** | **Sequences (5'-3')** | **Product length (bp)** | **Accession numbers** |
| --- | --- | --- | --- |
| *β-actin* | F：TGCGGGACATCAAGGAGAAG  R：AGTTGAAGGTGGTCTCGTGG | 216 | XM_003357928.4 |
| *Claudin-1* | F：AGATTTACTCCTACGCTGGT  R：GCACCTCATCATCTTCCAT | 249 | NM_001244539.1 |
| *Occludin* | F：ACAGACTACACAACTGGCGG  R：TCATCAGCAGCAGCCATGTA | 242 | XM_005672525.3 |
| *ZO-1* | F：ACTTGTCAGCTCAGCCAGTC  R：ACAGGCCTCAGAAATCCAGC | 82 | XM_021098896.1 |
| *TXNRD1* | F：GATTTAACAAGCGGGTCATGGT  R：CAACCTACATTCACACACGTTCCT | 94 | NM_214154.3 |
| *TXNRD2* | F：GCTACAGGACAGAAAGGTCAAG  R：TTCCACAGCACCTTCGATATG | 72 | NM_001168702.1 |
| *SELS* | F：GAGGCAGAGGCACCTGGAT  R：CTGCTAAAGCCTCCTGTCGTTT | 77 | NM_001164113.1 |
| *SELI* | F：GATGGTGTGGATGGAAAGCAA  R：GCCATGGTCAAAGAGTTCTCCTA | 72 | XM_021085655.1 |
| *SEPHS2* | F：TGGCTTGATGCACACGTTTAA  R：TGCGAGTGTCCCAGAATGC | 72 | NM_001093735.1 |
| *SELO* | F：GAAGATGCGGAAGAAGTTG  R：AAGTAGAAGGTGTTGGTGAA | 120 | NM_001201431.1 |
| *SELX* | F：ATCCCTAAAGGCCAAGAATCATC  R：GGCCACCAAGCAGTGTTCA | 111 | NM_001097460.1 |
| *SEPP1* | F：AACCAGAAGCGCCAGACACT  R：TGCTGGCATATCTCAGTTCTCAGA | 113 | NM_001134823.1 |
| *DIO1* | F：CATGGCCAAGAACCCTCACT  R：CCAGAAATACTGGGCACTGAAGA | 70 | NM_001001627.1 |
| *DIO2* | F：CATTCTTGGTCTCAGTCTCTA  R：GTTCTCGCCTCTCATTCAT | 168 | NM_001001626.2 |
| *GPX1* | F：GATGCCACTGCCCTCATGA  R：TCGAAGTTCCATGCGATGTC | 80 | NM_214201.1 |
| *GPX2* | F：TTCTACGACCTCAGTGCTA  R：CGAAGACAGGATGCTCATT | 328 | NM_001115136.1 |
| *GPX3* | F：TGCACTGCAGGAAGAGTTTGAA  R：CCGGTTCCTGTTTTCCAAATT | 80 | NM_001115155.1 |
