# Supplementary figures and images for: Selenium-enriched Cardamine violifolia improves growth performance with potential regulation of intestinal health and antioxidant function in weaned pigs
Source: Front Vet Sci. 2022 Aug 11;9:964766. doi: 10.3389/fvets.2022.964766 (PMC9403540; doi:10.3389/fvets.2022.964766)

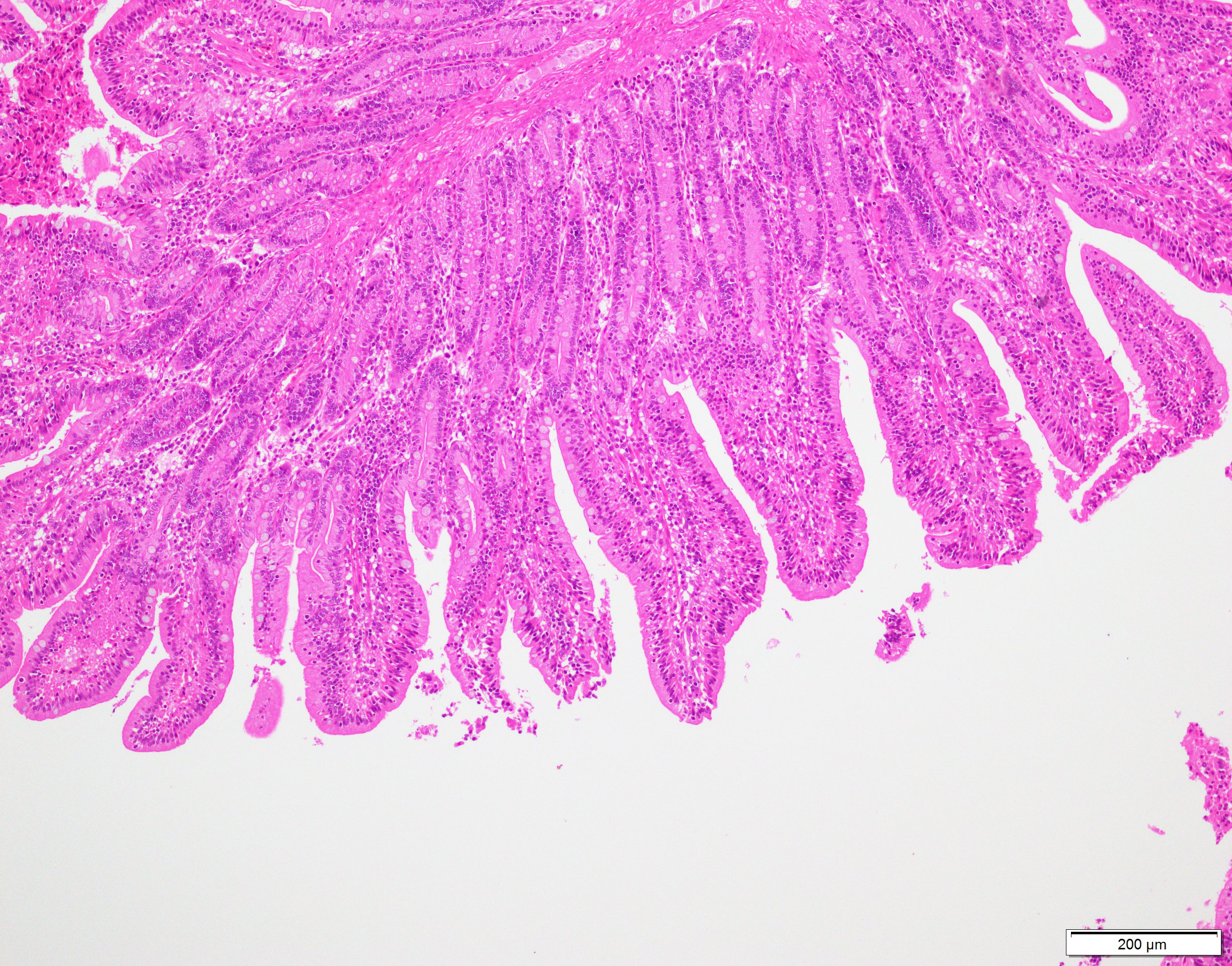

Supplement: Supplementary file 2 [file Data_Sheet_1.ZIP › Source data/Figure 1/Representative images/Ctrl.jpg]

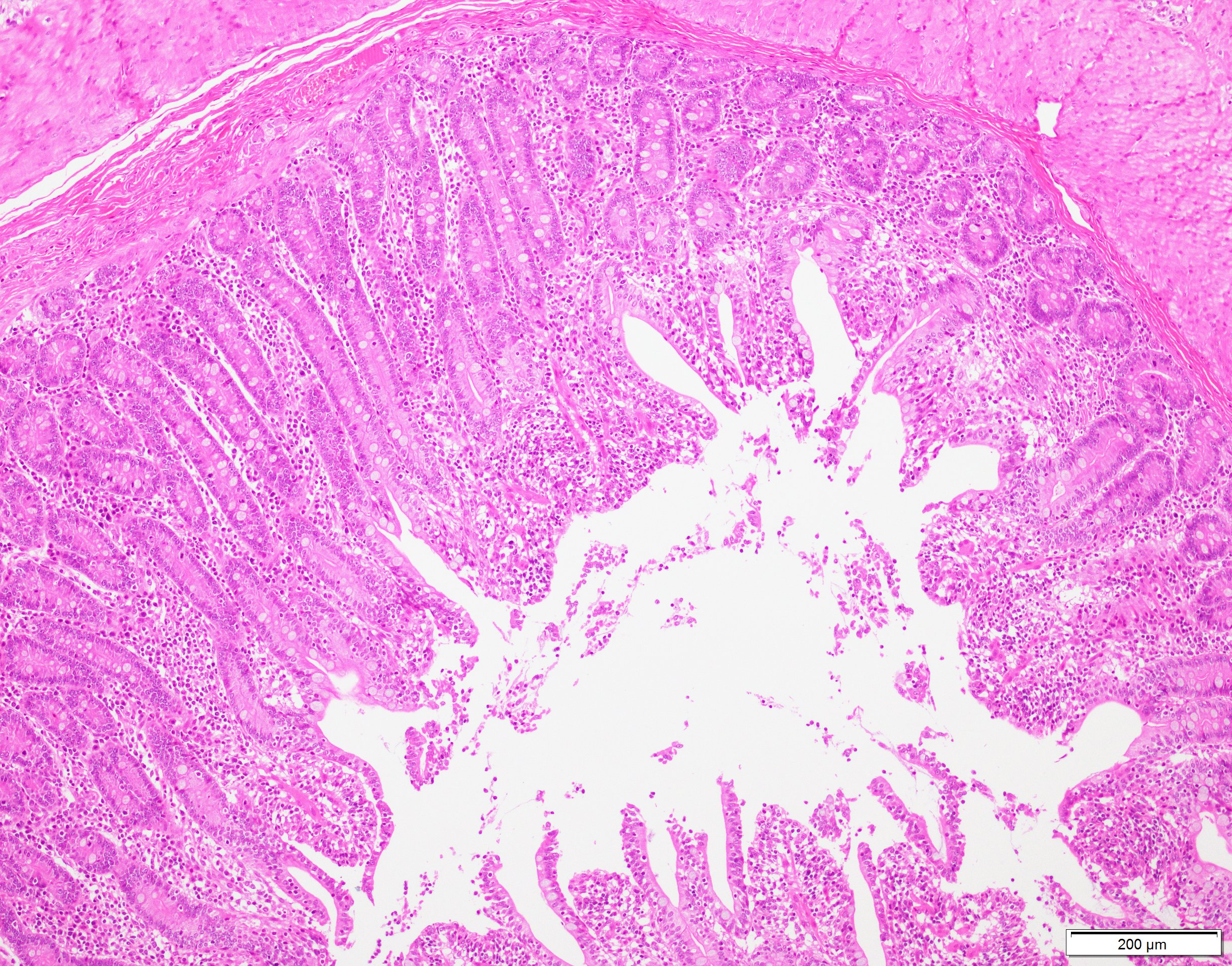

Supplement: Supplementary file 2 [file Data_Sheet_1.ZIP › Source data/Figure 1/Representative images/SEC.jpg]

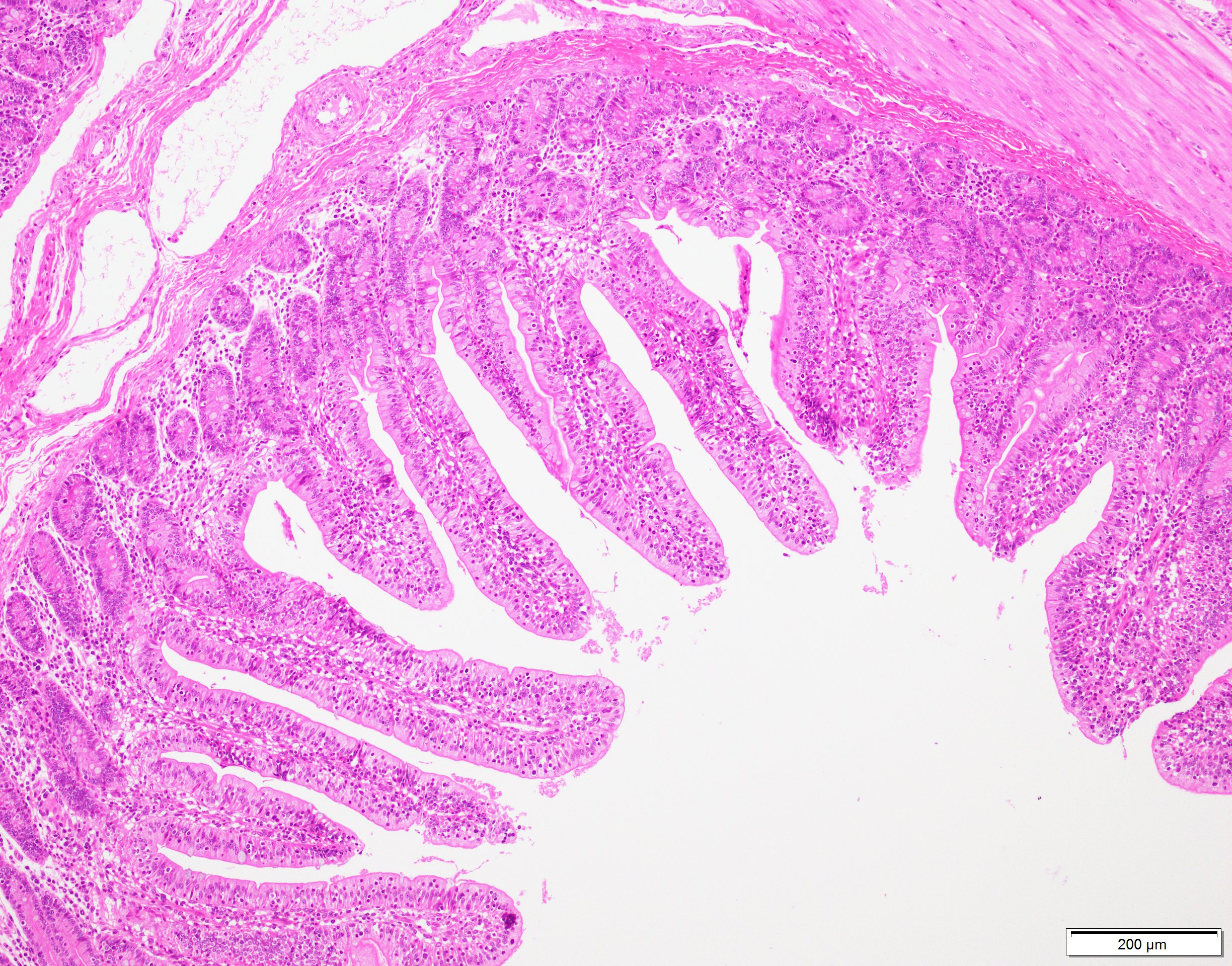

Supplement: Supplementary file 2 [file Data_Sheet_1.ZIP › Source data/Figure 1/Representative images/SEC+SEY.jpg]

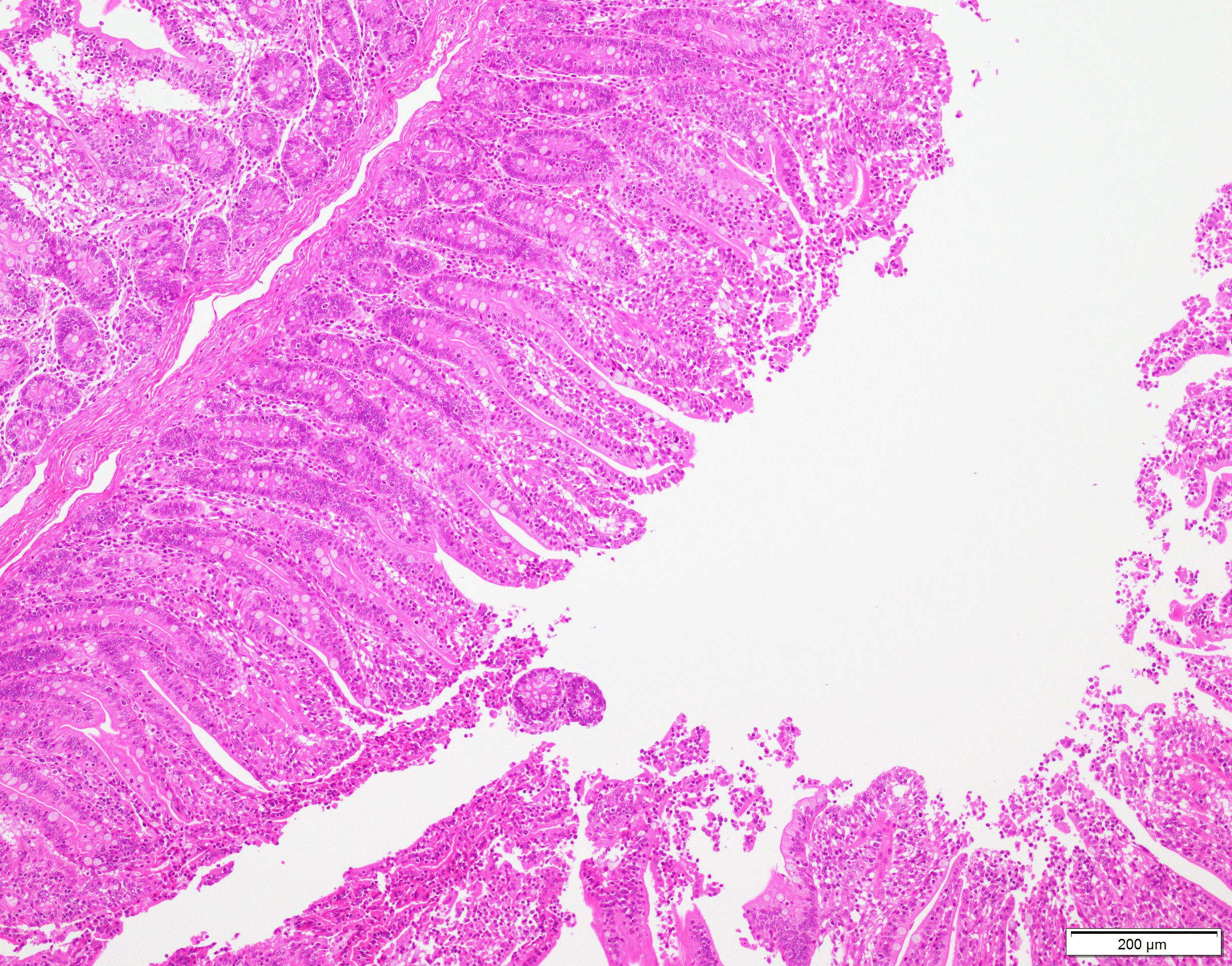

Supplement: Supplementary file 2 [file Data_Sheet_1.ZIP › Source data/Figure 1/Representative images/SEY.jpg]

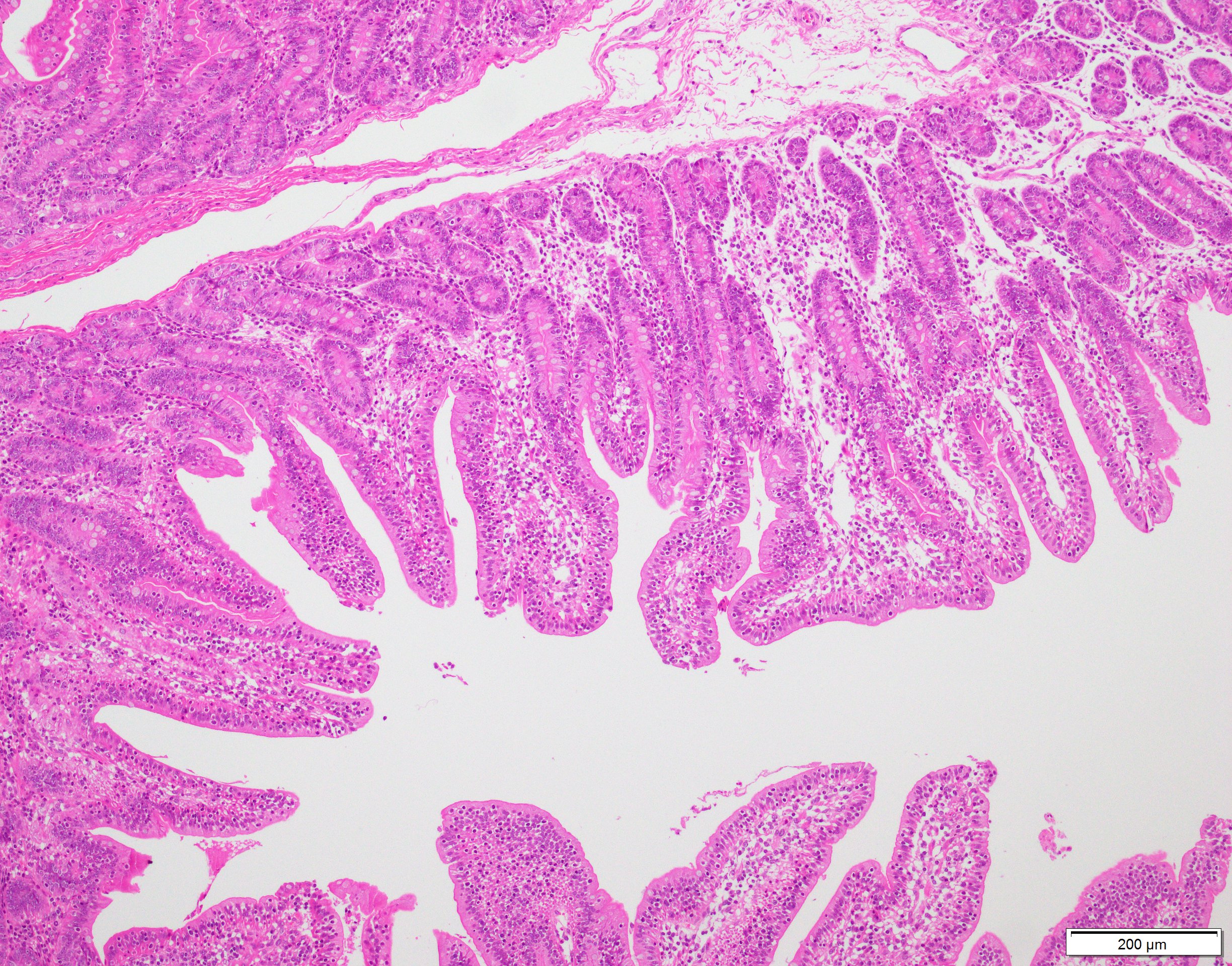

Supplement: Supplementary file 2 [file Data_Sheet_1.ZIP › Source data/Figure 1/Representative images/SS.jpg]
